# Neural Substrates of Interactive Musical Improvisation: An fMRI Study of ‘Trading Fours’ in Jazz

**DOI:** 10.1371/journal.pone.0088665

**Published:** 2014-02-19

**Authors:** Gabriel F. Donnay, Summer K. Rankin, Monica Lopez-Gonzalez, Patpong Jiradejvong, Charles J. Limb

**Affiliations:** Department of Otolaryngology-Head and Neck Surgery, Johns Hopkins University School of Medicine, Baltimore, Maryland, United States of America; National Research & Technology Council, Argentina

## Abstract

Interactive generative musical performance provides a suitable model for communication because, like natural linguistic discourse, it involves an exchange of ideas that is unpredictable, collaborative, and emergent. Here we show that interactive improvisation between two musicians is characterized by activation of perisylvian language areas linked to processing of syntactic elements in music, including inferior frontal gyrus and posterior superior temporal gyrus, and deactivation of angular gyrus and supramarginal gyrus, brain structures directly implicated in semantic processing of language. These findings support the hypothesis that musical discourse engages language areas of the brain specialized for processing of syntax but in a manner that is not contingent upon semantic processing. Therefore, we argue that neural regions for syntactic processing are not domain-specific for language but instead may be domain-general for communication.

## Introduction

Music and language are both complex systems of auditory communication that rely upon an ordered sequence of sounds to convey meaning, yet the extent to which they share formal, functional and neural architecture is an ongoing topic of debate. Music and language differ substantially in their use of pitch, rhythmic metrical structure, the form and function of their syntactic structures, and their ability to convey semantic precision and propositional thought [1]–[3]. Researchers have argued that music follows a system of syntactic rules akin to spoken language whose neural processing is linked to activity in the inferior frontal gyrus (Broca's area and its right hemisphere homologue [4]). However, due to the inherently abstract nature of music, scientists and musicologists have been unable to reconcile how the concept of musical semantics relates to language semantics or to determine the neural basis for any purported relationship between the two[5].

Fundamentally, music and language are both complex hierarchical combinatorial systems in which smaller units (notes in music and morphemes in language) can be combined to produce an infinite number of more complex structures [3], [6]–[8]. It is the generative capacity of music and language that allows each to serve as a means of communication between individuals, whether the content is aesthetic and emotional or pragmatic and semantic. This basic commonality between music and language raises the possibility of a shared network of neural structures that subserve these generative, combinatorial features. Patel and colleagues [9] articulated a similar idea as the ‘shared syntactic resource hypothesis’, whereby shared neural substrates serve syntactic processing in both language and music. Here we argue that musical communication involves an exchange of ideas that is not based on traditional notions of semantics, but instead on syntactic attributes.

Despite the large number of studies that have investigated the neural basis of music perception, none have examined the interactive and improvisational aspects of musical discourse [10], [11]. Improvisation, in jazz specifically, has drawn theoretical comparisons to linguistic discourse [12]–[14]. In the stylistic convention of trading fours, jazz musicians spontaneously exchange improvised material in four measure segments. This exchange is akin to a musical conversation in which the participants introduce novel melodic material, respond to each other's ideas, and elaborate or modify those ideas over the course of a performance. There are no formal rules for ‘successful’ trading fours in jazz, and this musical dialogue can take many forms [15]–[17]. Up to this point, our understanding of how auditory communication is processed in the brain has been entirely approached through the framework of spoken language, but trading fours provides a means of investigating the neurobiology of interactive musical communication as it occurs outside of spoken language.

## Materials and Methods

### Subjects

Eleven right-handed, healthy, male musicians (age range 25–6 years, mean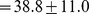 s.d.) with normal hearing participated in the study. All subjects were professional musicians that were highly proficient in jazz piano performance. None of the subjects had any history of neurologic, auditory or psychiatric disorders. Informed consent was obtained in writing for all subjects, and the research protocol was approved by the Johns Hopkins School of Medicine Institutional Review Board.

### Improvisation Paradigms

Two block-design imaging paradigms were used to assess interaction between two expert jazz pianists during improvisation. The first paradigm, Scale, assessed brain activity during a highly constrained task of minimal musical complexity. The second paradigm, Jazz, examined musical interaction of greater complexity and ecological validity. Subject A played a non-ferromagnetic MIDI keyboard in the fMRI scanner while Subject B played a MIDI keyboard in the control room. Both musicians heard their own and each other's performance along with a pre-recorded rhythm section accompaniment over headphones.

In Scale, subjects were cued to perform one of two tasks. During the control task (Scale – Control), Subject A and Subject B alternated playing a D Dorian scale in quarter notes with their right hand. During the interactive task (Scale – Improv), Subject A and Subject B took turns improvising four measure phrases (trading fours). For all experiments, Subject A was always the scanner subject and always played first in all musical exchanges. Subject B was always one of the two authors (G.F.D or C.J.L), both highly trained jazz musicians. Improvisation was restricted to continuous quarter notes in D Dorian, one octave. Musicians were instructed to listen and respond to each other's musical ideas. The tempo of the recorded accompaniment was 96 beats per minute. There were five 40-second blocks of each task separated by 20-second rest blocks for a total time of 10 minutes (each block consisted of four four-measure phrases, for a total of 16 measures). In Jazz, subjects were cued to perform one of two tasks. During the control task (Jazz – Control), Subject A and Subject B alternated playing four-measure segments of a novel jazz composition that subjects memorized prior to scanning (“Tradewinds” (Figure S2), composed by G.F.D. and C.J.L.). During the interactive task (Jazz – Improv), Subject A and Subject B traded fours. Improvisation was unrestricted melodically and rhythmically, but the subjects were instructed to play monophonically and to listen and respond musically to each other's playing. The tempo of the recorded accompaniment was 144 beats per minute. There were seven 60-second blocks of each task separated by 30-second rest blocks for a total time of 20.5 minutes (each block consisted of nine four-measure phrases, for a total of 36 measures). In both paradigms, Subject A always played first, and the control and experimental blocks were presented in pseudorandom order.

### Procedure

During scanning, subjects used a custom-built non-ferromagnetic piano keyboard (MagDesign, Redwood, CA) with thirty-five full-size plastic piano keys. The keyboard had Musical Instrument Digital Interface (MIDI) output, which was sent to a Macintosh Macbook Pro laptop computer running the Logic Pro 9 sequencing environment (Apple Inc., Cupertino, CA). The MIDI input triggered high-quality piano samples using the Logic EXS24 sampler plug-in. Piano sound output was routed back to the subject via in-ear electrostatic earspeakers (Stax, Saitama, Japan). In the scanner the piano keyboard was placed on the subject's lap in supine position, while their knees were elevated with a bolster. A double mirror placed above the subject's eyes allowed visualization and proper orientation of the keys during performance. Subjects were instructed to use only their right hand during scanning and were monitored visually to ensure that they did not move their head, trunk, or other extremities during performance. The subjects lay supine in the scanner without mechanical restraint. In addition to the electrostatic earspeakers, subjects wore additional ear protection to minimize background scanner noise. Ear speaker volume was set to a comfortable listening level that could be easily heard over the background scanner noise. A parallel signal path was used for the keyboard outside the scanner, which was an Oxygen USB MIDI controller (M-Audio, Los Angeles, CA) that was programmed to trigger an electric piano sample from Logic, so that each musician was represented by a distinct musical sound. The non-scanner subject (Subject B) was able to hear Subject A via an M-Audio Studiophile AV40 free-field monitor. See Figure S1 for a diagram of the experimental equipment setup.

### Scanning Parameters

All studies were performed at the F.M. Kirby Research Center for Functional Brain Imaging at the Kennedy Krieger Institute of Johns Hopkins University. Blood oxygen level dependent imaging (BOLD) data were acquired using a 3-Tesla whole-body scanner (Philips Electronics, Andover, MA) using a standard quadrature head coil and a gradient-echo EPI sequence. The following scan parameters were used: TR = 2000 ms, TE = 30 ms, flip-angle = 90 u, 64664 matrix, field of view 220 mm, 26 parallel axial slices covering the whole brain, 6 mm thickness. Four initial dummy scans were acquired during the establishment of equilibrium and discarded in the data analysis. For each subject, 300 volumes were acquired during the Scale paradigm and 630 volumes were acquired during the Jazz paradigm. BOLD images were preprocessed in standard fashion, with spatial realignment, normalization, and smoothing (9 mm kernel) of all data using SPM8 software (Wellcome Trust Department of Imaging Neuroscience, London, U.K.).

### Functional Neuroimaging Analysis

fMRI data analysis was performed by entering individual subject data from all eleven subjects into a group-matrix. Fixed-effects analyses were performed with a corrected threshold of 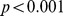 and random-effects analyses were performed with a corrected threshold of 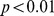 for significance. Contrast analyses were performed for activations and deactivations across all conditions (Scale – Control vs. Scale – Improv and Jazz – Control vs. Jazz – Improv). Areas of activation during Improv were identified by applying inclusive masking (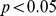 corrected) to contrasts for [ Improv > Control ] with contrasts for [ Improv > Rest ], 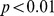 corrected, in order to identify true activations. Areas of deactivation during improvisation were revealed by applying inclusive masking of contrasts for [ Control > Improv ] with the contrasts of [ Rest > Improv ], 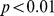 corrected to identify true deactivations.

### Behavioral Analysis

We applied signal processing methods to analyze the MIDI piano output obtained during fMRI scanning. The purpose of this analysis was to quantitatively evaluate the musical interaction between Subject A and Subject B. Several measures from the MIDI Toolbox [18] were used to classify and compare the four conditions and the phrases traded between A subjects and B subjects, including, note density, pitch class distribution, pitch class transitions, duration distribution, duration transitions, interval distribution, interval transitions, melodic complexity, and self-organizing maps of key.

Melodic complexity (available as *complebm* function in MIDI Toolbox [19]) was derived from Eerola and North's melodic expectancy model which focuses on tonal and accent coherence, the amount of pitch skips, and contour self-similarity. Melodic complexity can be described as the extent to which a melody violates a listener€s expectations; the stronger the violation, the more complex the melody. The model used in calculating melodic complexity has been coined expectancy-based model [20] of melodic complexity because it was designed to objectively model perceptual processes which underlie human listeners' musical expectations and complexity judgements. This function creates melodic predictability values which have been found to correspond to the predictability [19] and similarity ratings [21] given by listeners in experiments. The melodic complexity function is an aggregate of several other functions found in the MIDI Toolbox including, pitch class distribution (weighted by note duration), tonal stability (the correlations of the pitch-class distribution with each of the 24 Krumhansl-Kessler profiles [22], entropy of the interval distribution (the distribution of intervals using 25 components spaced at semitone distances spanning one octave weighted by note durations and metrical position [23]), mean interval size, syncopation (a measure of deviation from the anticipated, regular beat pattern [24]), rhythmic variability (the standard deviation of the durations), and rhythmic activity (the number of notes per second). A complete explanation of the features in these functions can be found in Eerola, Toiviainen & Krumhansl [19] or Eerola, et al. [21].

## Results

### Behavioral Results

**Figure 1:**
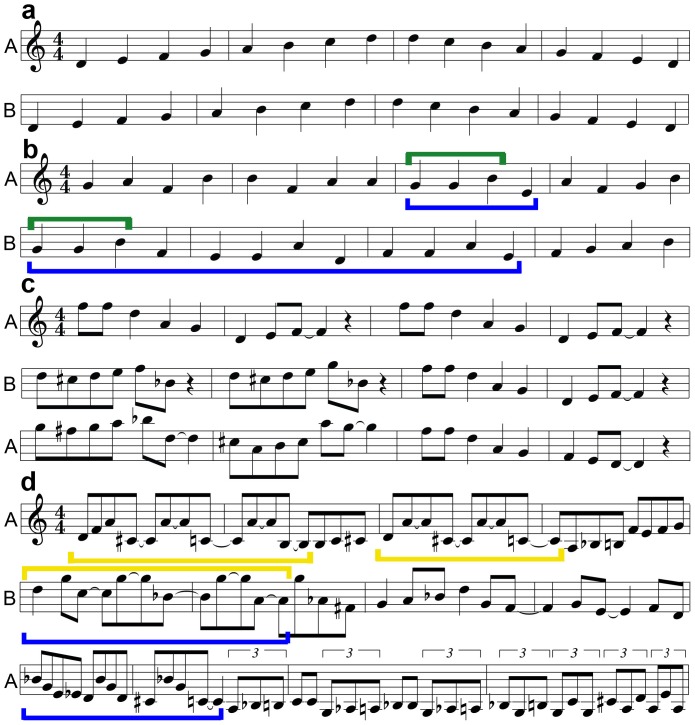
Examples of trading fours between one Subject A (A) and one Subject B (B), for each condition. In the Scale – Control condition (**a**), Subject A and Subject B traded a one octave, ascending and descending, D Dorian scale. In the Scale – Improv condition (**b**), Subject A and Subject B traded four measure improvised phrases; improvisation was heavily restricted to continuous, monophonic quarter notes in the key of D Dorian. In the Scale paradigm, there were five 40-second blocks of each task separated by 20-second rest blocks for a total time of 10 minutes. In the Jazz – Control condition (**c**), Subject A and Subject B traded four measures of a memorized jazz composition, “Tradewinds”. In the Jazz – Improv condition (**d**), Subject A and Subject B traded four measure improvisations; the only restriction in this improvisation condition was monophony (one note at a time). For the Jazz paradigm, there were seven 60-second blocks of each task separated by 30-second rest blocks for a total time of 20.5 minutes. Examples of interactions during trading are highlighted by colored brackets: green = repetition, blue = motivic development, and red = transposition.

We analyzed all MIDI output using qualitative music-theoretical criteria, which allowed us to demonstrate the frequency and degree to which specific types of improvisation occurred (e.g., contour imitation, contour inversion, melodic imitation, motivic development, repetition, and transposition; Figure 1, Figure S3). Most of the quantitative measures showed a significant difference between the conditions and a significant correlation between the paired phrases of Subject A and Subject B. For the quantitative analysis, eight phrase pairs were removed (1%) because one subject performed the task incorrectly. The number of notes played during the Scale – Control and Scale – Improv conditions were identical (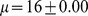 s.d.), the mean number of notes per subject for the Jazz – Control condition and Jazz – Improv condition were 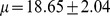 s.d. and 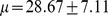 s.d. notes per block, respectively.

**Figure 2:**
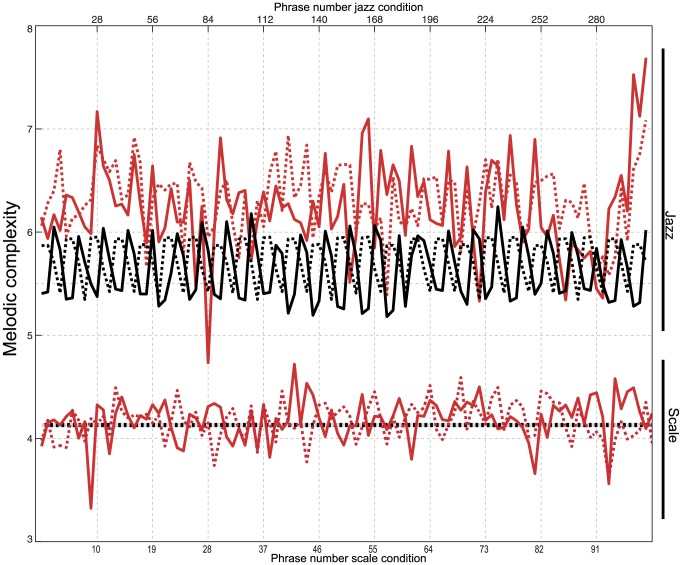
Melodic complexity is plotted for each phrase (Scale) or every third phrase (Jazz). Data from the A subjects (solid line) and the B subjects (dotted line) are shown sequentially as a continuous line. Control conditions are plotted in black and Improv conditions are plotted in red. In the condition Scale – Control (lower black line) melodic complexity was low and constant for both A subjects and B subjects, as expected (mean 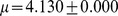 s.d., 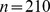). In Scale – Improv (lower red lines) the melodic complexity values change for each phrase, (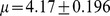 s.d., 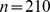). The two Jazz conditions are plotted in the upper portion of the graph; the melodic complexity is plotted for every third phrase, shown on the upper x-axis. For the Jazz – Control (upper black lines) condition, melodic complexity changed in a repetitive pattern because the same melody was being traded between the two musicians (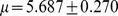 s.d., 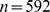). For Jazz – Improv (upper red lines), the melodic complexity values were higher (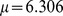) and significantly more variable (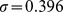 s.d.) than the other four conditions. A t-test was performed on the standard deviations which showed that data from the Jazz – Improv condition was significantly more variable than the other three conditions at 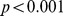.

Melodic complexity was calculated for each phrase played by A subjects and B subjects (Figure 2). The melodic complexity values are scaled between 0 and 10 (higher value indicates higher melodic complexity). We used melodic complexity in order to compare our data for improvised conditions to our data for control conditions. We were primarily interested in the relative differences between conditions rather than the absolute numerical value of the melodic complexity assessment, in order to show specifically that improvised melodies were more complex and more variable than control melodies, and that musicians were interacting with each other, as evidenced by the similarities in findings for paired phrases. A one-way analysis of variance on the melodic complexity values revealed a main effect of condition [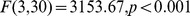]. Post-hoc pairwise comparisons (t-tests) showed that the melodic expectancy values for each condition were significantly different from one another at 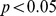. For the Scale – Control condition, which was anticipated to have the lowest degree of melodic complexity, the mean melodic complexity score was 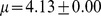 s.d., 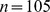 for A subjects and 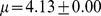 s.d., 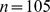 for B subjects. For the Scale – Improv condition, where the musical exchange had no rhythmic variability (all notes were quarter notes) and the exchange was limited to a one octave D Dorian scale, melodic complexity was significantly higher (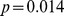) than for the Scale – Control condition (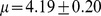 s.d., 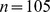 A subjects, 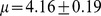 s.d., 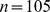 B subjects). The Jazz – Control condition, which consisted of a twelve bar blues melody in D Dorian, had a significantly higher melodic complexity (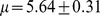 s.d., 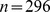 A subjects, 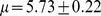 s.d., 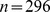 B subjects) than either of the Scale conditions (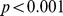), which is consistent with the expanded pitch range and rhythmic variability of this condition. The Jazz – Improv condition, in which interaction was unrestricted, had the highest melodic complexity of all the conditions which was significant at 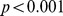 (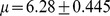 s.d., 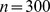 A subjects, 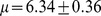 s.d., 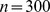 B subjects).

Several measures from the MIDI Toolbox [18] were used to quantify and compare the phrases that were traded between Subject A and Subject B because this parameter is an indication of the musical interaction, which was truly the most critical aspect of this study (i.e., the pitch class distribution for each phrase from each A subject was correlated with the pitch class distribution for the corresponding phrase from the B subject). Using cross-correlation, most measures showed a significant correlation between the paired phrases of the two musicians. These results are displayed in Table 1. We also examined the melodic complexity of the phrase pairs. Because the melodic complexity scores for the Scale – Control condition were identical, the cross correlation was perfect (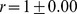 s.d.). For the Jazz – Control condition, the musicians (Subject A and Subject B) were significantly correlated with each other (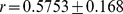 s.d.; Figure 2). The Improv conditions also showed positive but weaker correlation between the two musicians (Scale – Improv 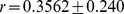 s.d.; Jazz – Improv 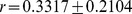 s.d.), as anticipated due to the variability of the improvised conditions in comparison to the control conditions. These correlations reveal that despite the higher level of melodic complexity and higher variability demonstrated by the musicians during improvisation, phrase pairs were related to one another both qualitatively and quantitatively. These findings strongly support the notion that the improvised material was both spontaneous and interactive in nature between the two musicians.

**Table 1:** Quantitative MIDI analysis of phrase pairs.

Condition	Duration		Duration 2		Pitch		Pitch 2		Interval		Interval 2	
	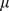	%	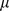	%	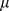	%	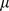	%	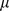	%	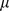	%
Scale Contol n = 105	1	100	1	100	1	100	1	100	1	100	1	100
Scale Improv n = 105	1	100	1	100	.7921	100	.3463	92.38	.465	66.67	.1915	75.24
Jazz Contol n = 296	.6763	56.76	.4094	79.39	.6954	80.41	.4116	85.14	.523	65.2	.4425	99.66
Jazz Improv n = 300	.6842	58	.4639	80.66	.4626	38.33	.1548	41	.5071	68.33	.1916	74.33

Table 1 shows the mean correlation coefficient (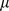) between six different musical features for each phrase for Subject A and Subject B averaged over all subjects; we also show the percentage of phrase pairs that were significantly correlated at 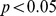. **Duration** is the distribution of note durations (nine components on a logarithmic scale). **Duration 2** is the 2nd order distribution of durations (transitions from one note to the next). **Pitch** is the distribution of pitch classes weighted by note duration. **Pitch 2** is the 2nd order pitch class distribution weighted by note duration (transitions from one pitch to the next). **Interval** is the distribution of intervals using 25 components spaced at semitone distances spanning one octave weighted by note durations and metrical position [23]. **Interval 2** is the 2nd order interval distribution (distribution of interval dyads weighted by note durations).

### Functional Neuroimaging Results

**Figure 3:**
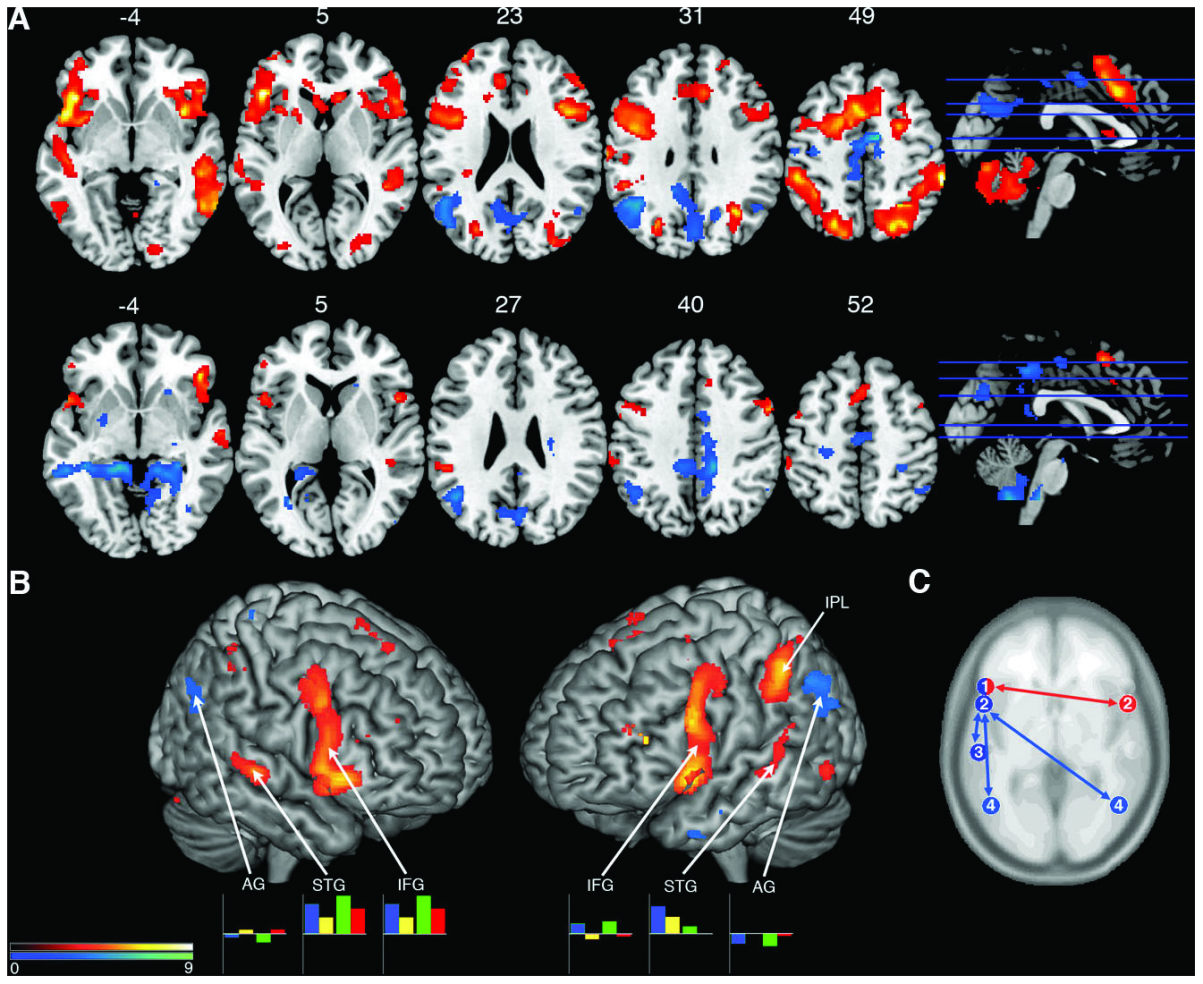
Visualization of neuroimaging results. (**A**) Axial slice renderings of activations and deactivations associated with improvisation during Scale (top) and Jazz (bottom) paradigms. In both paradigms, improvisation was associated with bilateral activations in language and sensorimotor areas and lateral prefrontal cortex and bilateral deactivations in angular gyrus. Activations were identified through inclusive masking of the contrast for [ Improv > Control ] with the contrast for [ Improv > Rest ], and deactivations were identified through inclusive masking of the contrast for [ Control > Improv ] with the contrast for [ Rest > Improv ]. Sagittal sections show axial slice location. Labels refer to axial slice z-plane in MNI space. (**B**) 3D surface projection of activations and deactivations associated with improvisation as determined by a conjunction analysis across paradigms. Bar graphs indicate percent signal change at cluster maxima (with y-axis scaled from -1 to 1) for Scale – Control (blue), Scale – Improv (yellow), Jazz – Control (green), and Jazz – Improv (red). Scale bars indicate t-score values for both A and B. (**C**) Selected results from functional connectivity analysis. Red arrows indicate correlated activity, blue arrows indicate anti-correlated activity. 1 = IFG pTri, 2 = IFG pOp, 3 = STG, 4 = AG.

Results from both paradigms were largely congruent at both the fixed- and random-effect levels of analysis. Table 2 shows stereotactic coordinates in MNI space for local maxima and minima for selected activations and deactivations that reached our statistical threshold for significance (see Table S2 for the unabridged list of activations and deactivations). Contrast and conjunction analyses between Improvised and Control conditions were performed at the random effects level for both Scale and Jazz paradigms. In comparison to memorized, non-improvised exchange, improvised exchange was characterized by intense activation in Broca's area (inferior frontal gyrus, pars opercularis and pars triangularis; Brodmann areas 45 and 44) and Wernicke's area (posterior STG; Brodmann area 22), two classical perisylvian language regions (Figure 3). In addition, the right hemisphere homologues of both of these areas were also active, more so on the right than the left for the posterior STG (Table 2). Improvisation was also associated with strong bilateral deactivation of the angular gyrus, an area that has been identified as a cross-modal center for semantic integration in numerical, linguistic, and problem-solving processing, among other things [25]–[27]. Functional connectivity analysis of language regions and contralateral homologues during spontaneous exchange in Jazz revealed significant positive correlations between right IFG left IFG, as well as a pattern of anti-correlated connectivity for bilateral IFG STG and left IFG bilateral AG (Table 3).

**Table 2:** Selected local maxima and minima of activations and deactivations during interactive improvisation.

Activations	Scale	Jazz
Region	BA	Left Hemisphere					Right Hemisphere					Left Hemisphere					Right Hemisphere				
		t-score	x	y	z	Cluster Size	t-score	x	y	z	Cluster Size	t-score	x	y	z	Cluster Size	t-score	x	y	z	Cluster Size
Perisylvian Language Areas																					
IFG-PTr	45	10.85	−44	28	8	8756	6.51	52	22	2	3059	3.6	−54	34	12	82	6.07	50	24	0	426
IFG-POp	44	6.03	−60	8	14	8756	5.34	56	16	0	3059	3.02	−60	12	24	10	5.23	50	14	4	426
STG	22	3.17	−56	−6	−2	515	4.69	66	−46	20	41	3.87	−54	−46	12	13	8.65	64	−24	2	176
Sensorimotor																					
Motor																					
SMA	6	6.6	−6	18	48	8756	3.95	6	22	58	8756	5.73	0	16	54	201	3.66	8	24	54	201
Temporal																					
ITG	20	5.17	−56	−62	−10	251	8.77	58	−58	−10	12074	-	-	-	-	-	2.82	52	−52	−24	1
MTG	37	5.04	−54	−42	2	515	9.42	58	−38	2	12074	4.68	−46	−24	−2	20	2.92	42	−52	18	1
Parietal																					
IPL	2, 40	10.38	−42	−36	40	2872	10.20	58	−36	50	12074	3.8	−48	−42	58	220	3.26	58	−36	54	8
SPL	7	7.73	−24	−70	48	2872	6.95	20	−72	50	12074	3.43	−12	−80	50	3	-	-	-	-	-
SMG	2, 40	7.08	−2	22	40	8756	3.86	12	60	32	14	4.89	−50	−38	28	220	2.78	62	−26	46	1
Occipital																					
Mid OG	18, 19	6.03	−26	−72	24	2872	3.34	44	−72	−16	12074	-	-	-	-	-	3.33	42	−74	22	5

Deactivations	Scale	Jazz
Region	BA	Left Hemisphere					Right Hemisphere					Left Hemisphere					Right Hemisphere				
		t-score	x	y	z	Cluster Size	t-score	x	y	z	Cluster Size	t-score	x	y	z	Cluster Size	t-score	x	y	z	Cluster Size
Prefrontal Cortex																					
MFG	6	3.21	−46	28	36	6	3.78	24	48	6	27	−3.29	−38	20	52	10	−3.41	40	38	32	14
SFG	8, 10	6.38	−18	6	52	8756	4.99	18	46	36	64	−3.2	−18	58	20	16	−3.23	26	−8	68	16
Precentral Gyrus	6, 44	−3.95	−22	−18	74	22	−4.68	44	−10	56	138	−5.47	−22	−18	64	985	−5.05	54	6	18	33
Other																					
Angular Gyrus	39	−4.64	−48	−52	26	1045	−4.78	50	−52	34	179	−5.17	−44	−60	26	474	−3.17	62	−58	26	46
Precuneus	31	−4.26	−8	−56	18	1854	−4.29	18	−64	26	1854	−5.95	−14	−54	68	985	−2.78	6	−58	46	1

Coordinates described according to the Montreal Neurological Institute system were obtained from a random effects analysis of contrasts of [ Improv > Control ] masked inclusively with [ Improv > Rest ] and [ Control < Improv ] masked inclusively with [ Rest < Improv ]. Threshold was 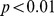 for contrasts and 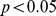 for masks.

Abbreviations: IFG, inferior frontal gyrus; PTr, pars triangularis; POp, pars opercularis; STG, superior temporal gyrus; SMA, supplementary motor area; ITG, inferior temporal gyrus; MTG, middle temporal gyrus; IPL, inferior parietal lobule; SPL, superior parietal lobule; SMG, supramarginal gyrus; OG, occipital gyrus; MFG, medial frontal gyrus; SFG, superior frontal gyrus.

**Table 3:** Functional connectivity.Correlations and anti-correlations in BOLD activation revealed by analysis with the Functional Connectivity Toolbox in SPM8.

	Region 1	Region 2	beta	t-score	p-unc
Correlated	R IFG pOp	R IFG pTri	0.13	2.4	.019
	R IFG pOp	L IFG pTri	0.14	2.06	.033
Anti-Correlated	R IFG pOp	R STG	−0.18	−3.6	.002
	L IFG pOp	R STG	−0.25	−2.95	.007
	L IFG pOp	L STG	−0.24	−2.65	.012
	L STG	R STG	−0.21	−2.54	.015
	R IFG pTri	R STG	−0.18	−2.52	.015
	R IFG pOp	L STG	−0.18	−2.47	1.6E-2
	R IFG pTri	L STG	−0.15	−2.32	.021
	L IFG pOp	L AG	−0.14	−1.98	.038
	L IFG pOp	R AG	−0.14	−1.95	0.04
	L IFG pTri	L AG	−0.14	−1.89	.044

Activations and deactivations were also observed in sensorimotor areas and prefrontal cortex. In neocortical sensory areas, increased activity was observed bilaterally in the middle and superior occipital gyrus, supramarginal gyrus, inferior and middle temporal gyrus and inferior and superior parietal lobule. There was also intense bilateral activation across the supplementary motor area (SMA) associated with improvised communication in comparison to memorized exchange. Spontaneous musical exchange was associated with bilateral activation of dorsolateral prefrontal cortex (DLPFC) as well as strong deactivation in the dorsal prefrontal cortex bilaterally, concentrated along the superior frontal gyrus and the middle frontal gyrus. A conjunction analysis for both Scale and Jazz showed congruency across paradigms for activations in IFG, STG, SMA and DLPFC bilaterally as well as the left inferior parietal lobule and medial temporal gyrus (Figure 3B–C).

## Discussion

This study represents the first effort, to our knowledge, to examine the neural substrates of generative, interactive musical behavior. Our results reveal that improvised musical communication, in comparison to memorized exchange, leads to intense engagement of left hemispheric cortical areas classically associated with language, as well as their right hemispheric homologues. Trading fours was characterized by activation of the left IFG (Broca's area) and left posterior STG (Wernicke's area), areas that are known to be critical for language production and comprehension as well as processing of musical syntax [28]–[30]. In addition to left perisylvian structures, right hemisphere homologues of Broca's and Wernicke's areas were also activated. The right IFG is associated with the detection of task relevant cues [31] such as those involved in the identification of salient harmonic and rhythmic elements. The right STG has been implicated in auditory short-term memory [32], consistent with the maintenance of the preceding musical phrases in short-term memory while trading fours. Especially relevant are previous findings that suggest involvement of Broca's area and its right hemisphere homologue in syntactic processing for both music and speech [4], [33] and involvement of Wernicke's area in harmonic processing [34], given the production of melodically-, rhythmically-, and harmonically-related musical sequences we observed within phrase pairs.

Although many neuroimaging studies have examined speech production and perception, only one has examined the perception and generation of spontaneous linguistic discourse. In a study of spoken conversation involving the evaluation of congruence between question-answer pairs, functional activation was observed in Broca's and Wernicke's areas and their right hemisphere homologues, the cerebellum, and DLPFC [35]. The overlap in the neural activation observed in that study and the present report may be attributable to the topic maintenance of in-the-moment information required in both linguistic conversation and musical interaction. These shared linguistic-musical results are consistent with the “shared syntactic integration resource hypothesis” which proposes that music and language representation in the brain share a common neural network for syntactic operations, but not necessarily semantic ones [3]. While there are specific grammatical categories (e.g., nouns in language) that have no direct correlate in music, there are conceptual parallels like hierarchical structure (e.g., words are grouped into phrases which are grouped into higher-level phrases; and notes are grouped into motifs which are grouped into phrases which are further grouped into sections) to account for the observed functional activation for both linguistic and musical tasks. It should be emphasized that our experiment was not designed to analyze the modulation of neural activity during a trading fours block (for example, the difference between listening or responding within each block), and further study is needed to examine this important issue.

We observed robust bilateral deactivation of the parietal cortex, specifically the angular gyrus, during trading fours. Given this area's implication in semantic processing of auditory and visual linguistic stimuli and the production of written language and music, the correlation between deactivation of the angular gyrus and improvisation may be indicative of the lesser role semantic processing has in moment-to-moment recall and improvisatory musical generation whereby only musical syntactic information is exchanged and explicit meaning is intangible and possibly superfluous. Functional deactivation during musical communication in regions associated with angular gyrus-mediated semantic processing for language raise important questions with regard to the application of linguistic definitions of semantics to music. Theories of musical semantics have disagreed significantly, with some positing that music can communicate a variety of meanings-from differing emotions (e.g., happy vs. sad) [36]–[38] to extramusical associations (typified, for example, by the similarities between an object such as a staircase and a musical structure such as an ascending scale[36], [39]–and others discussing its capacity to communicate quite specific propositional thoughts [40]. Such contrasting views obscure the notion, however, that meaning in music is fundamentally context-specific [41] and imprecise, thereby differing wholly from meaning in natural language (which aims at referential specificity) [42]. Our findings of angular gyrus deactivation may shed light on this debate. Deactivations in angular gyrus during goal-directed tasks have been hypothetically attributable to the interruption of task-free semantic and conceptual processes that results from the manipulation of acquired knowledge about the world. Musical communication as represented by trading fours is a type of task that is both perceptual (musical information is physically presented in the sensory stimulus) and conceptual (melodic, rhythmic and harmonic ideas are explicitly related to ongoing perceptual events). The significant deactivations observed in angular gyrus activity during improvised exchange compared to memorized exchange strongly suggest that spontaneous musical communication is not dependent upon natural language areas involved in semantic cognition, such as the angular gyrus, but soley upon acoustic-phonologic-analysis areas [43], as observed in posterior STG. Furthermore, this study underscores the need for a broader definition of musical semantics that balances organized hierarchical structure (conveyed through melody, rhythm and harmony) with in-the-moment instantiations of novel ideas that are semantically imprecise.

While our data show medial frontal deactivation in medial SFG and dorsal MFG, and bilateral activation of the precentral gyrus and DLPFC, Limb & Braun [44] found lateral deactivation in DLPFC and lateral orbitofrontal cortex (LOFC) paired with frontal activation in the medial prefrontal cortex (MPFC); DLPFC deactivation was attributed to the disinhibited state of “flow” which is subjectively reported by musicians while improvising. In the present study, however, the additional social context of trading fours may provide an explanation for the unexpected activation of DLPFC. Since the DLPFC has been linked to conscious self-monitoring of behavior, an increased BOLD response in this area is expected in a social context. Additionally, the DLPFC has been associated with the on-line manipulation of information and response selection [45], suggesting a correlation between DLPFC activation and increased working memory demands while trading. In comparison to solo musical improvisation, there is greater expectation during a musical conversation that what is played will be melodically and or rhythmically related to the immediate antecedent musical phrase, placing potentially greater demands on working memory. This increased self-monitoring interpretation is substantiated by the fact that the right IFG was also active during trading, an area associated with response inhibition [31]. A further observation in this study was widespread activation of sensorimotor areas in both improvised paradigms. This enhanced activity may be indicative of a “primed” state as the musician prepares to execute unplanned ideas in a spontaneous context. We also observed deactivation in limbic and paralimbic structures, including the hippocampus, parahippocampal gyrus, posterior cingulate gyrus and temporal pole. Deactivation in the hippocampus, parahippocampal gyrus and temporal pole may be attributable to a positive affective response to improvisation, as deactivation of these structures has been associated with the experience of pleasure when listening to consonant music [4].

## Conclusion

The results presented here provide important insights into the neural overlap between music and language processing and support the view that these systems rely in part on a common network of prefrontal and temporal cortical processing areas. These results suggest strongly that these neural resources may not be domain-specific for spoken language, but rather domain-general for auditory communication more broadly. Furthermore, our study provides important evidence that parietal cortex structures involved in semantic processing for language are not involved in spontaneous musical exchange, suggesting a fundamental difference between how meaning is conveyed in music and language.

## Supporting Information

Figure S1 **Diagram of experimental equipment setup.** E-Prime software triggered audio stimuli, which were routed through a mixer to headphones for the subject in the scanner and experimenter in the control room, as well as an audio monitor. MIDI input from the musicians' MIDI keyboards triggered piano samples in Logic Pro, which were also routed through the mixer and heard by both A and B subjects.
(TIF)

Figure S2 **Tradewinds.** A musical composition written by GFD and CJL for this experiment. It was written in the style of a traditional 12-bar blues. All subjects memorized this composition prior to scanning and performed it during the Jazz – Control condition.
(TIF)

Figure S3 **Annotated excerpts from MIDI data illustrating examples of each category of interaction, percentage of trading pairs characterized by type of interaction.** a) The first phrase is ascending and ends on the dominant scale degree. The second phrase is descending and ends on the tonic. b) In the first phrase, the same melodic contour is repeated for three measures (two ascending notes followed by one descending note). In the second phrase, this melodic contour is repeated for three more measures, starting on different scale degrees. c) The first phrase ascends until the first beat of measure three, then descends to the end of measure four. The second phrase does the opposite, descending until the second beat of measure seven before ascending to the end of measure eight. d) The second phrase has nothing in common melodically with the first phrase. This excerpt is an example of the introduction of a novel idea during trading. e) The bracketed motif in the first phrase is inverted and transposed in the second phrase. f) The bracketed segment of the first phrase is imitated in the second phrase (but not exactly repeated–the arrows indicate notes that are identical, but the others deviate). g) The bracketed motif in the first phrase is developed in the second phrase. The original motif has three repeated notes followed by two descending notes. The response phrase begins with three repeated notes followed by two descending notes, but places a larger interval between the repeated and descending notes and adds an ascending interval at the end of the motif. This motif is subsequently repeated twice (although the second repetition is truncated by the end of the block). h) The bracketed segment of the first phrase is repeated exactly in the second phrase. i) The rhythm in the bracketed segment is repeated multiple times. j) The bracketed motif in the first phrase is repeated twice in the second phrase, but transposed upwards by one scale degree. Note: All excerpts are drawn from the Scale condition except for the Rhythmic Imitation example, which was from the Jazz condition.
(TIF)

Table S1
**All maxima and minima from contrast Improv – Control.** All coordinates are described according to the Montreal Neurological Institute system, and were obtained from a random effects analysis of contrasts of [Trade> Control ] masked inclusively with [Trade> Rest ] and [ Control < Trade] masked inclusively with [ Rest < Trade]. Threshold was 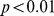 for contrasts and 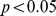 for masks.(TIF)

Audio S1 **Excerpt of one block of control condition in Scale task.**
(MP3)

Audio S2 **Excerpt of one block of experimental condition in Scale task.**
(MP3)

Audio S3 **Excerpt of one block of control condition in Jazz task.**
(MP3)

Audio S4 **Excerpt of one block of experimental condition in Jazz task.**
(MP3)
