# Supplementary figures and images for: Neural Substrates of Interactive Musical Improvisation: An fMRI Study of ‘Trading Fours’ in Jazz
Source: PLoS One. 2014 Feb 19;9(2):e88665. doi: 10.1371/journal.pone.0088665 (PMC3929604; doi:10.1371/journal.pone.0088665)

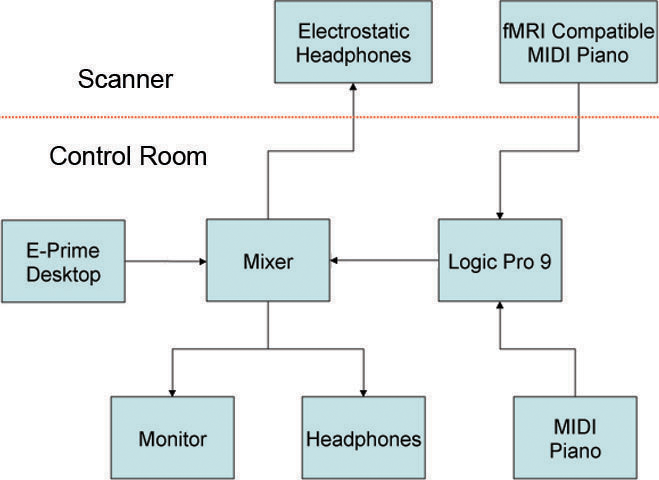

Supplement: Figure S1 — Diagram of experimental equipment setup. E-Prime software triggered audio stimuli, which were routed through a mixer to headphones for the subject in the scanner and experimenter in the control room, as well as an audio monitor. MIDI input from the musicians' MIDI keyboards triggered piano samples in Logic Pro, which were also routed through the mixer and heard by both A and B subjects. (TIF) [file pone.0088665.s001.tif]

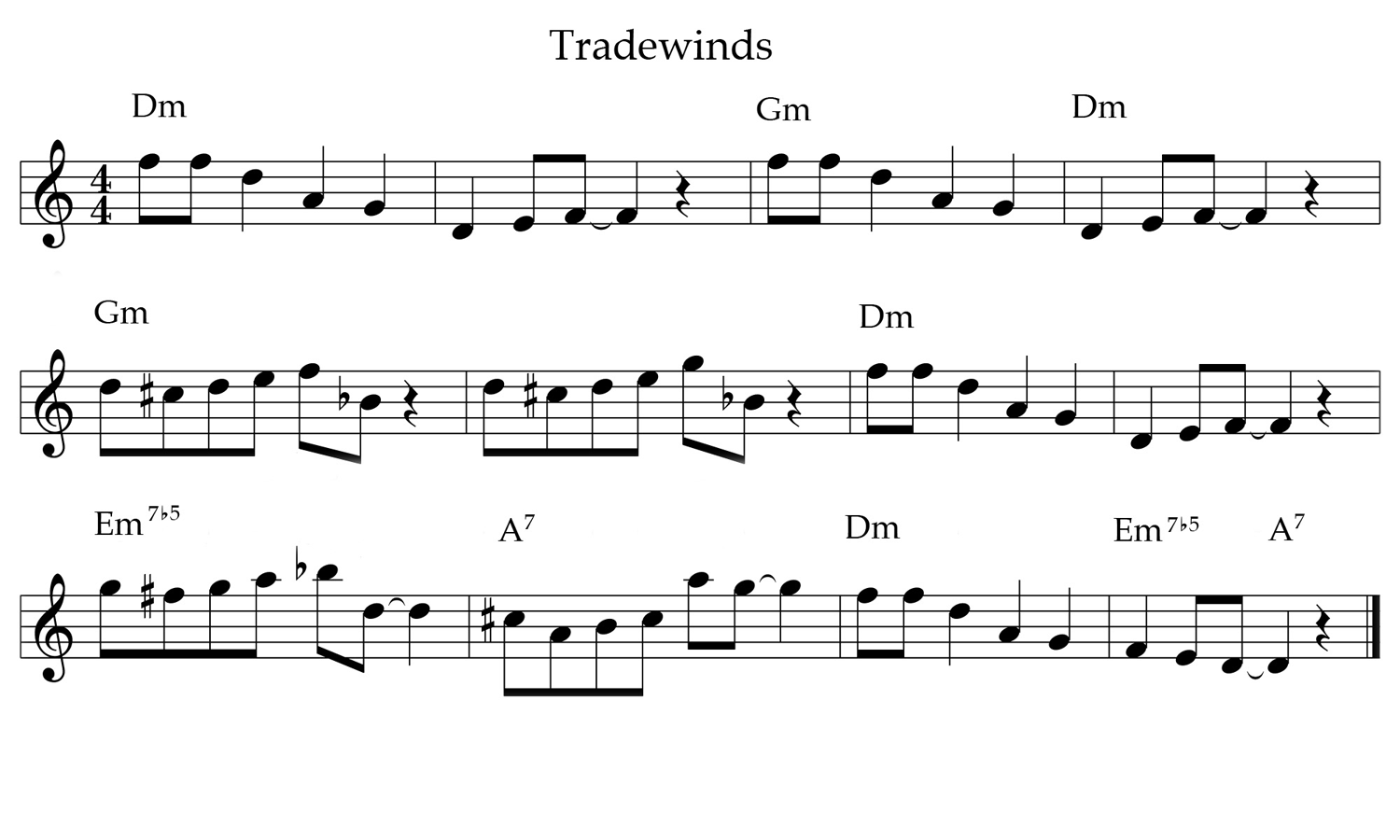

Supplement: Figure S2 — Tradewinds. A musical composition written by GFD and CJL for this experiment. It was written in the style of a traditional 12-bar blues. All subjects memorized this composition prior to scanning and performed it during the Jazz – Control condition. (TIF) [file pone.0088665.s002.tif]

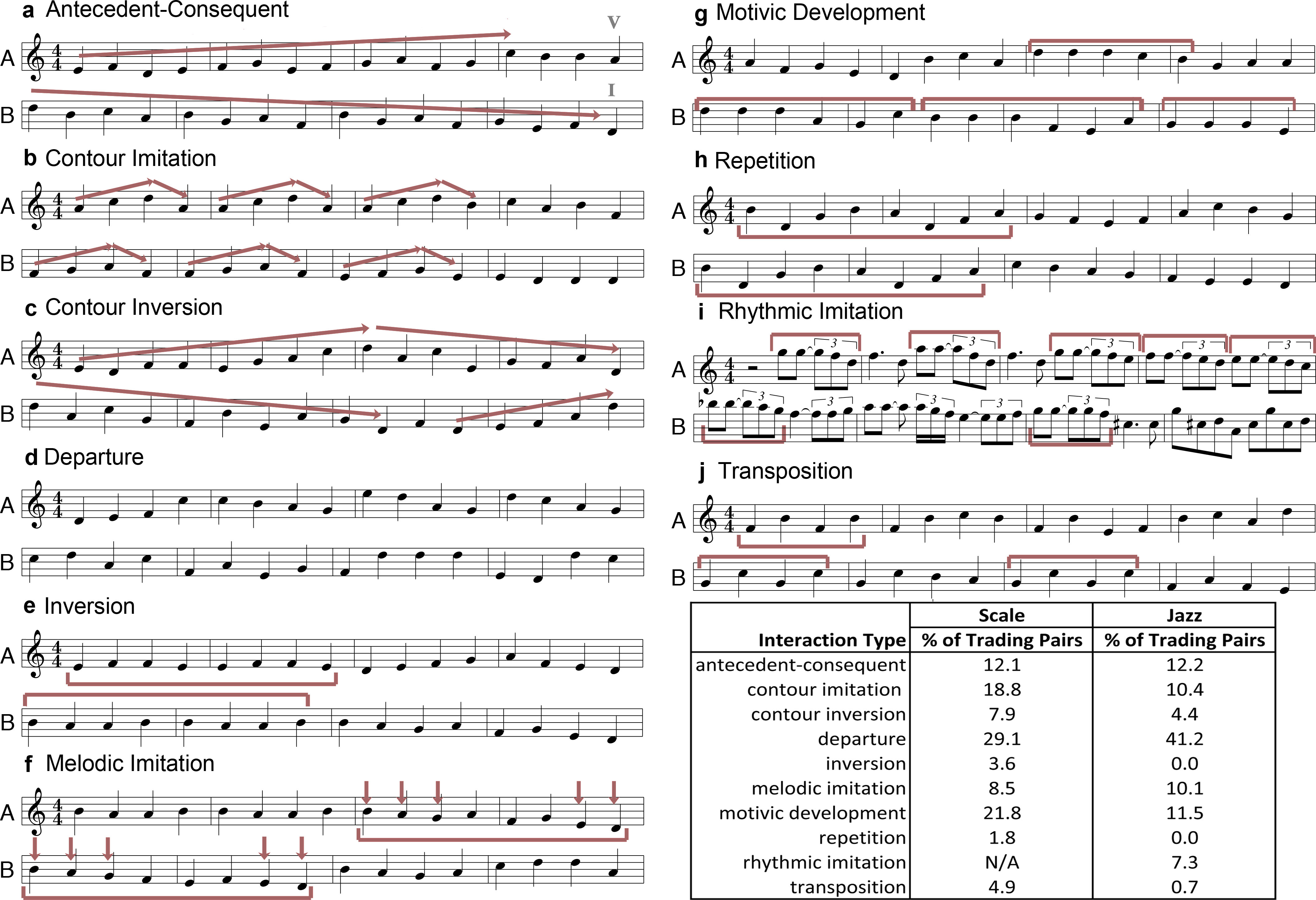

Supplement: Figure S3 — Annotated excerpts from MIDI data illustrating examples of each category of interaction, percentage of trading pairs characterized by type of interaction. a) The first phrase is ascending and ends on the dominant scale degree. The second phrase is descending and ends on the tonic. b) In the first phrase, the same melodic contour is repeated for three measures (two ascending notes followed by one descending note). In the second phrase, this melodic contour is repeated for three more measures, starting on different scale degrees. c) The first phrase ascends until the first beat of measure three, then descends to the end of measure four. The second phrase does the opposite, descending until the second beat of measure seven before ascending to the end of measure eight. d) The second phrase has nothing in common melodically with the first phrase. This excerpt is an example of the introduction of a novel idea during trading. e) The bracketed motif in the first phrase is inverted and transposed in the second phrase. f) The bracketed segment of the first phrase is imitated in the second phrase (but not exactly repeated–the arrows indicate notes that are identical, but the others deviate). g) The bracketed motif in the first phrase is developed in the second phrase. The original motif has three repeated notes followed by two descending notes. The response phrase begins with three repeated notes followed by two descending notes, but places a larger interval between the repeated and descending notes and adds an ascending interval at the end of the motif. This motif is subsequently repeated twice (although the second repetition is truncated by the end of the block). h) The bracketed segment of the first phrase is repeated exactly in the second phrase. i) The rhythm in the bracketed segment is repeated multiple times. j) The bracketed motif in the first phrase is repeated twice in the second phrase, but transposed upwards by one scale degree. Note: All excerpts are drawn from th [file pone.0088665.s003.tif]

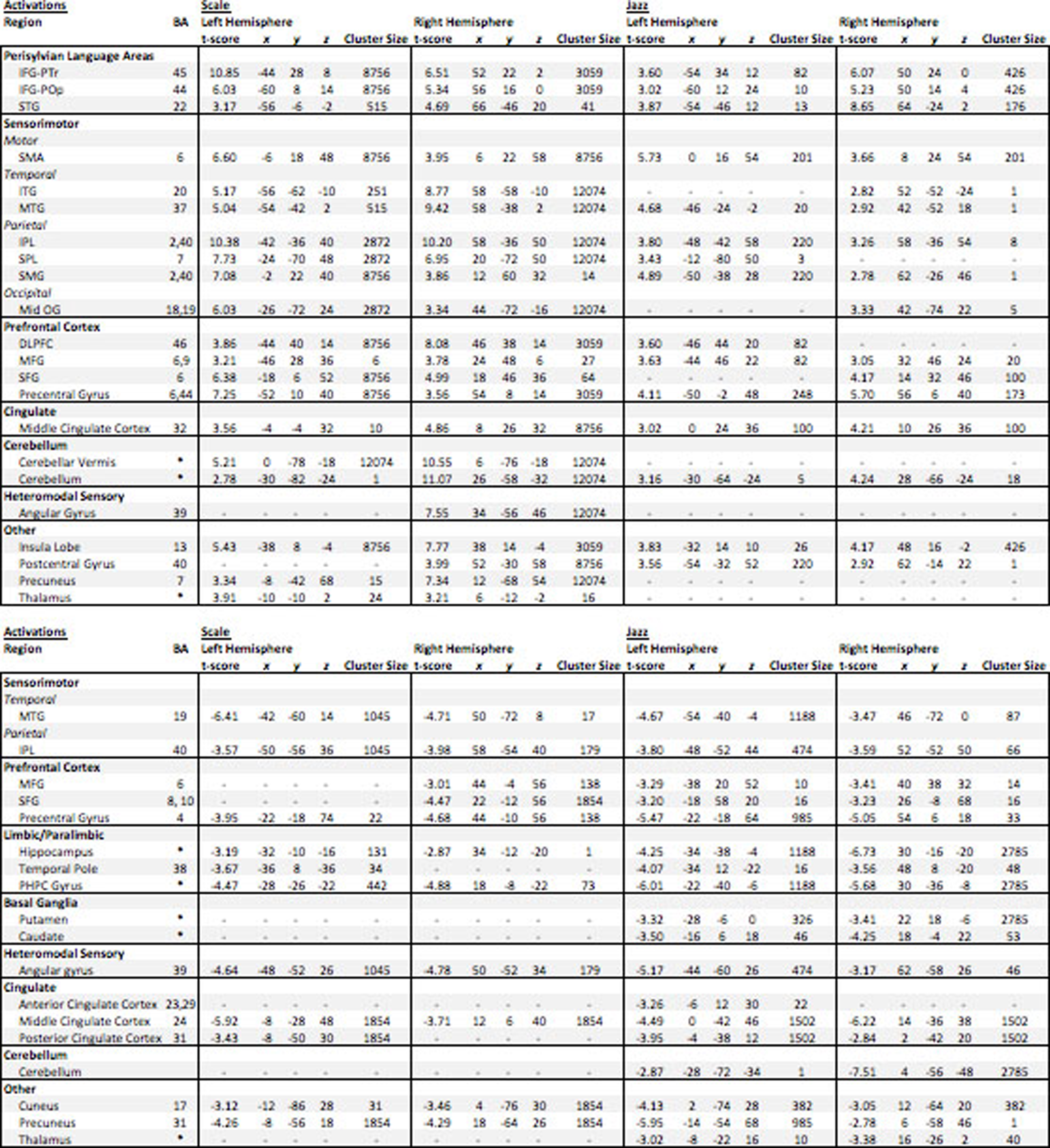

Supplement: Table S1 — All maxima and minima from contrast Improv – Control. All coordinates are described according to the Montreal Neurological Institute system, and were obtained from a random effects analysis of contrasts of [Trade> Control ] masked inclusively with [Trade> Rest ] and [ Control < Trade] masked inclusively with [ Rest < Trade]. Threshold was for contrasts and for masks. (TIF) [file pone.0088665.s004.tif]
